# Serial representation of items during working memory maintenance at letter-selective cortical sites

**DOI:** 10.1371/journal.pbio.2003805

**Published:** 2018-08-15

**Authors:** Ali Bahramisharif, Ole Jensen, Joshua Jacobs, John Lisman

**Affiliations:** 1 Department of Psychiatry, Academic Medical Center, Amsterdam, The Netherlands; 2 Radboud University, Donders Institute for Brain, Cognition and Behaviour, Nijmegen, The Netherlands; 3 Centre for Human Brain Health, School of Psychology, University of Birmingham, Birmingham, United Kingdom; 4 Department of Biomedical Engineering, Columbia University, New York City, New York, United States of America; 5 Volen Center for Complex Systems, Brandeis University, Waltham, Massachusetts, United States of America; Vanderbilt University, United States of America

## Abstract

A key component of working memory is the ability to remember multiple items simultaneously. To understand how the human brain maintains multiple items in memory, we examined direct brain recordings of neural oscillations from neurosurgical patients as they performed a working memory task. We analyzed the data to identify the neural representations of individual memory items by identifying recording sites with broadband gamma activity that varied according to the identity of the letter a subject viewed. Next, we tested a previously proposed model of working memory, which had hypothesized that the neural representations of individual memory items sequentially occurred at different phases of the theta/alpha cycle. Consistent with this model, the phase of the theta/alpha oscillation when stimulus-related gamma activity occurred during maintenance reflected the order of list presentation. These results suggest that working memory is organized by a cortical phase code coordinated by coupled theta/alpha and gamma oscillations and, more broadly, provide support for the serial representation of items in working memory.

## Author summary

In our daily lives, we often have to keep multiple items active in our working memory—for instance, a series of digits in a phone number. How does the brain perform this task? In 1995, Lisman and Idiart proposed that multi-item working memory was supported by a mechanism in which individual memory items are repeatedly activated in sequence. The temporal coordination of the activation was supported by coupled oscillatory brain activity in the theta/alpha (7–13 Hz) and gamma range (>30 Hz). Using intracranial recordings from the human brain, we now report experimental support for this scheme. In particular, when subjects remembered a list of working memory items, it activated theta/alpha oscillations. Simultaneously, the brain showed item-specific activations of gamma activity that appeared at a theta/alpha phase corresponding to the item’s position in the sequence. The work demonstrates that coupled cortical oscillations are important for coordinating the maintenance of multi-item working memory.

## Introduction

**Fig 1:**
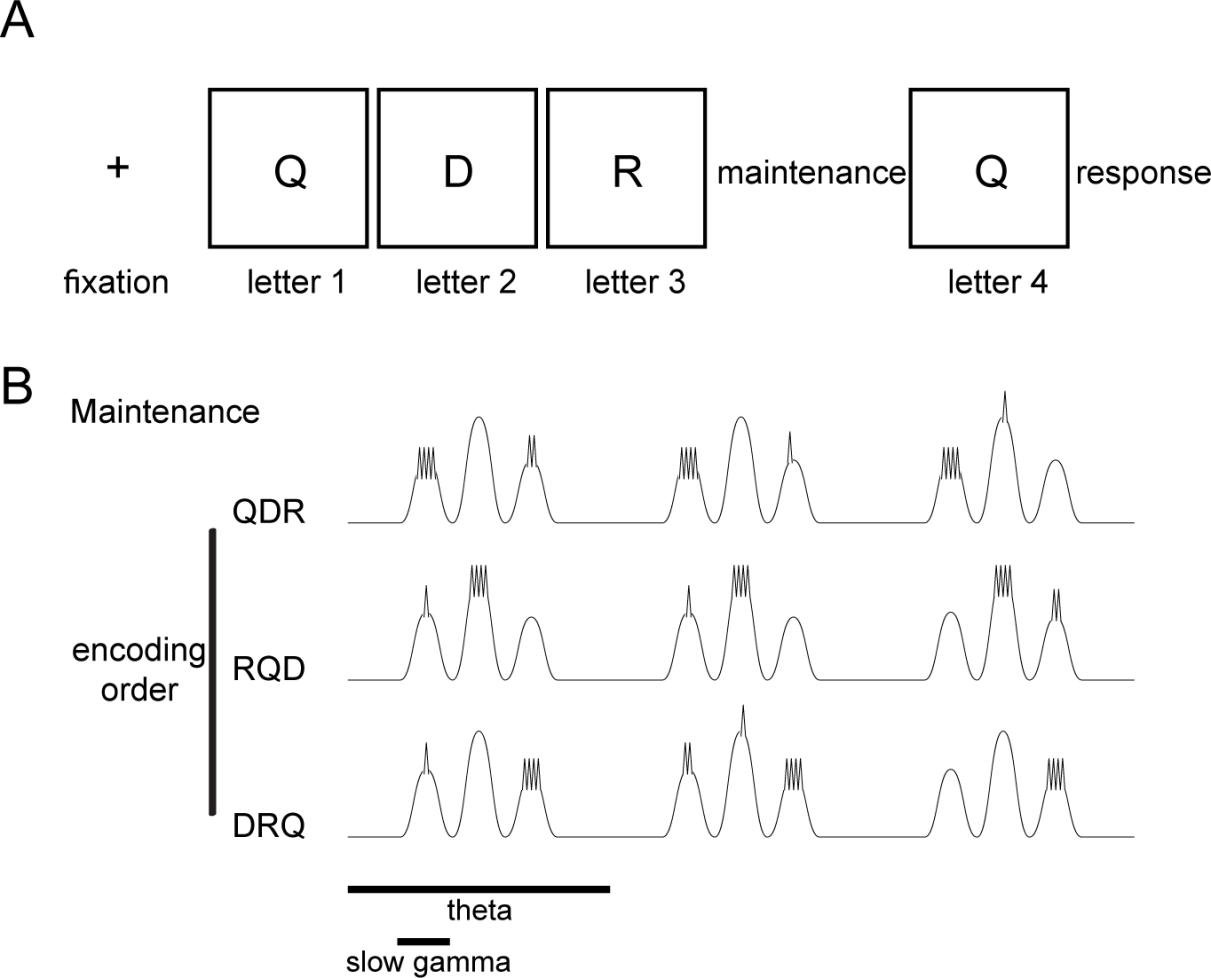
Schematic that illustrates the Sternberg task and an oscillatory model of multi-item WM. (A) Sternberg task with 3 letters each presented for 700 ms at an interstimulus interval of 275–350 ms. This was followed by a maintenance period of 2 s, followed by a 700 ms probe. (B) The dynamics predicted by the LIJ model for the WM maintenance on a site selective to “Q” with respect to 3 different positions on the list. Each theta/alpha cycle may contain many (5–8) gamma cycles (slow gamma), but only 3 are shown. The gamma cycle (slow gamma) at which maximal high-frequency firing occurs (and thus theta phase) corresponds to the order of item presentation. LIJ, Lisman/Idiart/Jensen; WM, working memory.

There is both functional and physiological evidence that working memory (WM) is mediated by a different neural system compared to long-term memory (LTM). The capacity of WM is much smaller than that of LTM [1]. Physiological data indicate that WM and LTM have different mechanisms: whereas LTM is supported by changes in synaptic weights, WM representations involve ongoing neuronal activity [2–7]; but see [8]. However, key aspects of the neural basis of WM are largely a mystery, and, in particular, we do not understand how a single network can store and then access multiple stimulus representations. Experiments using the Sternberg paradigm provided some insight into these processes (Fig 1A) by showing that when subjects answer whether a probe item is on a just-presented list, response time (RT) varied linearly with list length. This led to the suggestion that WMs are actively represented in a serial fashion and that these representations are sequentially scanned during recall [9].

A great deal of physiological work in humans and animals showed that the brain exhibits oscillations at multiple frequencies during cognition and, moreover, that these oscillations interact via a process called cross-frequency coupling (CFC) [10]. According to one model (Lisman/Idiart/Jensen [LIJ] model) [11,12], interacting oscillations in the gamma range (>30 Hz) (reviewed in [13]) and theta/alpha range (5–15 Hz) [14,15] organize multi-item WM. In this model, individual items are represented by the spatial pattern of neural activity within each gamma cycle; different list items are represented sequentially across consecutive gamma cycles, and this process then repeats on each theta cycle (Fig 1B). Such organization has been termed the theta–gamma code [11,16] and has been shown to be important in hippocampal processes that underlie encoding of sequences in both episodic memory [17] and in the replay of spatial paths [18–20].

Although it is now clear that the theta–gamma code has a critical role in hippocampal function, there is only limited information about the involvement of this representational scheme in neocortex [21–23]. Computational models based on the theta–gamma scheme show that this framework can explain subject reaction times in the Sternberg paradigm [11] and provide a physiologically plausible account of the ordered serial representation of memory items in WM. Although recordings of human neuronal oscillations have thus far indicated that these signals are involved generally in WM [15], these data have not provided specific support for any particular model of WM function. In particular, there is no evidence from human neocortex that individual WM items were represented on distinct phases of slow oscillations, as predicted by the theta–gamma model.

We studied the role of oscillations during WM maintenance by analyzing a set of cortical sites that supported memory for individual stimuli. We identified these sites because they demonstrated a level of stimulus-induced gamma band power that varied according to the identity of the stimulus that a subject viewed. Such content-dependent sites, primarily in the occipital and temporal lobes, were identified in recent studies during encoding [14,24], but their role in WM processes remained unclear. Here, we provide the first analysis of the properties of the signals during maintenance at the cortical sites that showed stimulus-specific during memory encoding in a WM task. The results showed a complex regulation of both theta/alpha and gamma band activity in which, notably, the relative amplitudes of these oscillations differed between encoding and maintenance. Further, during maintenance, these sites showed CFC between these oscillations, which we hypothesized indicated the existence of an oscillatory phase code that organized serial representation of WM items. We tested this idea by measuring activity at the sites that showed item-specific gamma band activity. Our results show that the theta/alpha phase of gamma bursts during WM maintenance depend on the order at which the items were presented during encoding. These results support the hypothesis that multi-item WM involves serial activation of memories, as first hypothesized by Sternberg [9] and physiologically implemented in the LIJ model (Fig 1B).

## Results

We analyzed intracranial recordings of electrocorticographic activity from subjects as they performed the Sternberg WM task (a total of 1,315 sites in 15 subjects). During the encoding phase of the task, 3 letters were presented sequentially, each visible for 700 ms followed by an interstimulus interval of 275–350 ms. After all list items were presented, there was a 2 s maintenance phase. Finally, a probe letter was presented, and subjects answered by indicating whether the probe was on the just-presented list (Fig 1A). The mean reaction times were 2,011 ± 412 ms, and the mean hit rates were 94.21% ± 1.28%. As in previous work [14,24,25], we first identified the “letter-selective” electrodes, which showed an increase in the amplitude of fast-gamma band activity (70–100 Hz; [14]) that varied depended on which letter was presented. We used a measure of mutual information (MI) in the gamma band to identify sensors with significant distinguishability of individual letters (*p* < 0.05, Bonferroni corrected; permutation test; see the Methods section Letter-selective sites). We then identified the “tuned” and “untuned” letters for each site. To do this, for each electrode, we first identified the specific pair of letters with the largest MI, and we then labeled the specific letters in this pair with higher and lower gamma representations, respectively, as tuned and untuned. As in [14], letter-selective sites were rare (14 sites in 9 subjects; 1 subject had 3 sites, and 3 subjects had 2 sites) and were located in the occipital and temporal lobes, mainly in the fusiform gyrus [26] (S1 Table and S1 Fig). We focused the subsequent analysis on these letter-selective sites. When considering the subjects with more than 1 letter-selective site, we found that these sites were selective to different letters. Therefore, we considered the 14 sites as statistically independent in the subsequent analysis. The relatively low number of letter-specific electrodes in this electrocorticographic dataset is not unexpected, given the limitations of these types of clinical recordings, in which electrode coverage is limited and, in particular, rarely includes coverage in sulci.

### Time-frequency analysis of power during encoding

**Fig 2:**
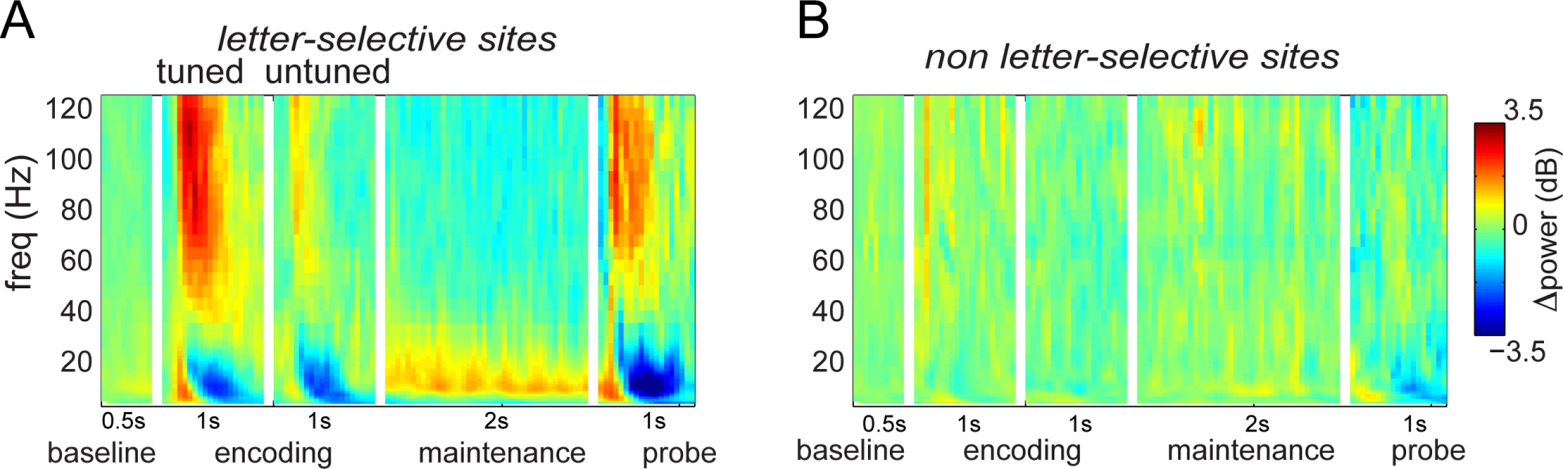
TFRs of power during encoding, maintenance, and probe. (A) Average TFRs of power at 14 letter-selective sites. From left to right, the panels of this plot show the neural responses to baseline, tuned and untuned letters, response during maintenance, and responses to the probe, respectively. During encoding, gamma activity is elevated by tuned letters; theta/alpha decreases by either the tuned or untuned letter. During maintenance, theta/alpha is elevated, and gamma is low. (B) Average TFRs of power at 14 non-letter-selective sites, which show little change during encoding or maintenance. Underlying data available at http://orion.bme.columbia.edu/jacobs/data/. TFR, time-frequency representation

We used time-frequency analysis to understand the role of oscillations in memory representations at these letter-specific sites. Fig 2 summarizes these results by averaging over 14 sites. The analysis confirmed that there was an increase in gamma power for letter-selective sites and only little modulation for other sites. We next examined how the low-frequency oscillations at these sites were affected by letter presentation (encoding). In contrast to the increased power in the gamma band, theta/alpha power transiently decreased in the 0.5–1 s interval after letter presentation (Fig 2A and 2B; two-tailed *t* test, *N* = 14; *p* = 0.0001 for average power at 7–13 Hz). Thus, theta/alpha and gamma power at letter-selective sites are differentially affected by letter presentation.

### Time-frequency analysis of power during maintenance

**Fig 3:**
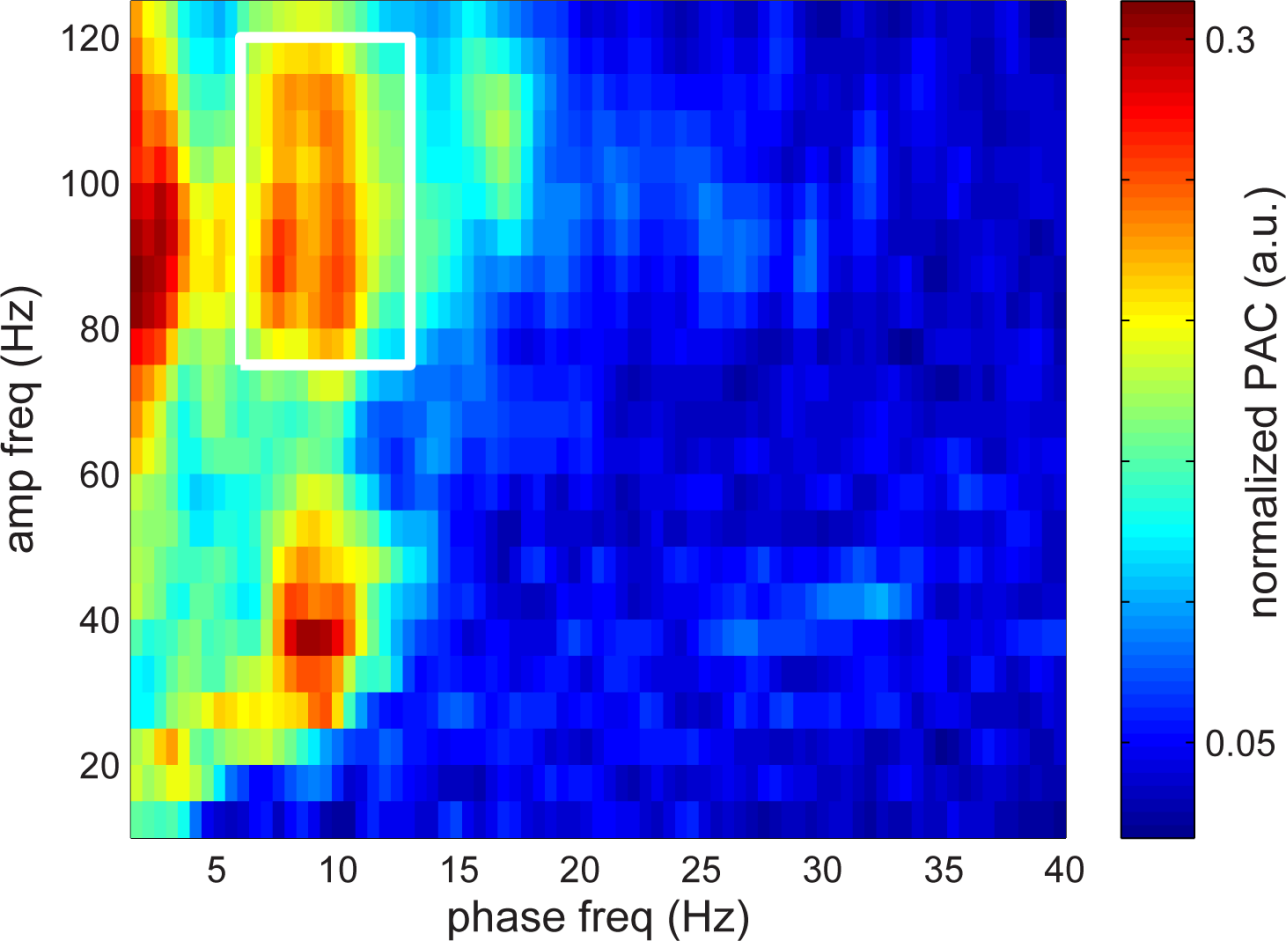
CFC during maintenance. The CFC was calculated by estimating the PAC for the data during the retention interval [27]. When combining all sensors, a coupling was observed between theta/alpha phase and gamma power. Note the coupling to slow gamma at 30–40 Hz and fast gamma activity at 75–120 Hz. Based on these results and the modulations in Fig 2, we focused the subsequent analysis on the theta/alpha band (7–13 Hz) and the gamma band (75–120 Hz). Underlying data available at http://orion.bme.columbia.edu/jacobs/data/. a.u., arbitrary unit; CFC, cross-frequency coupling; PAC, phase–amplitude coupling.

We next turned to the analysis of the electrophysiological activity during the maintenance phase of the Sternberg task. As a start, we calculated the average normalized phase–amplitude coupling (PAC) over all selective and nonselective sites during maintenance (Fig 3). This allowed us to preselect the frequency band of interest: the analysis revealed a peak in CFC between oscillatory phase in the theta/alpha band (7–13 Hz) and power in the high- (75–120 Hz) and low-gamma bands. As noted earlier, some previous work demonstrated continuous theta/alpha increases during both encoding and maintenance [15].

**Fig 4:**
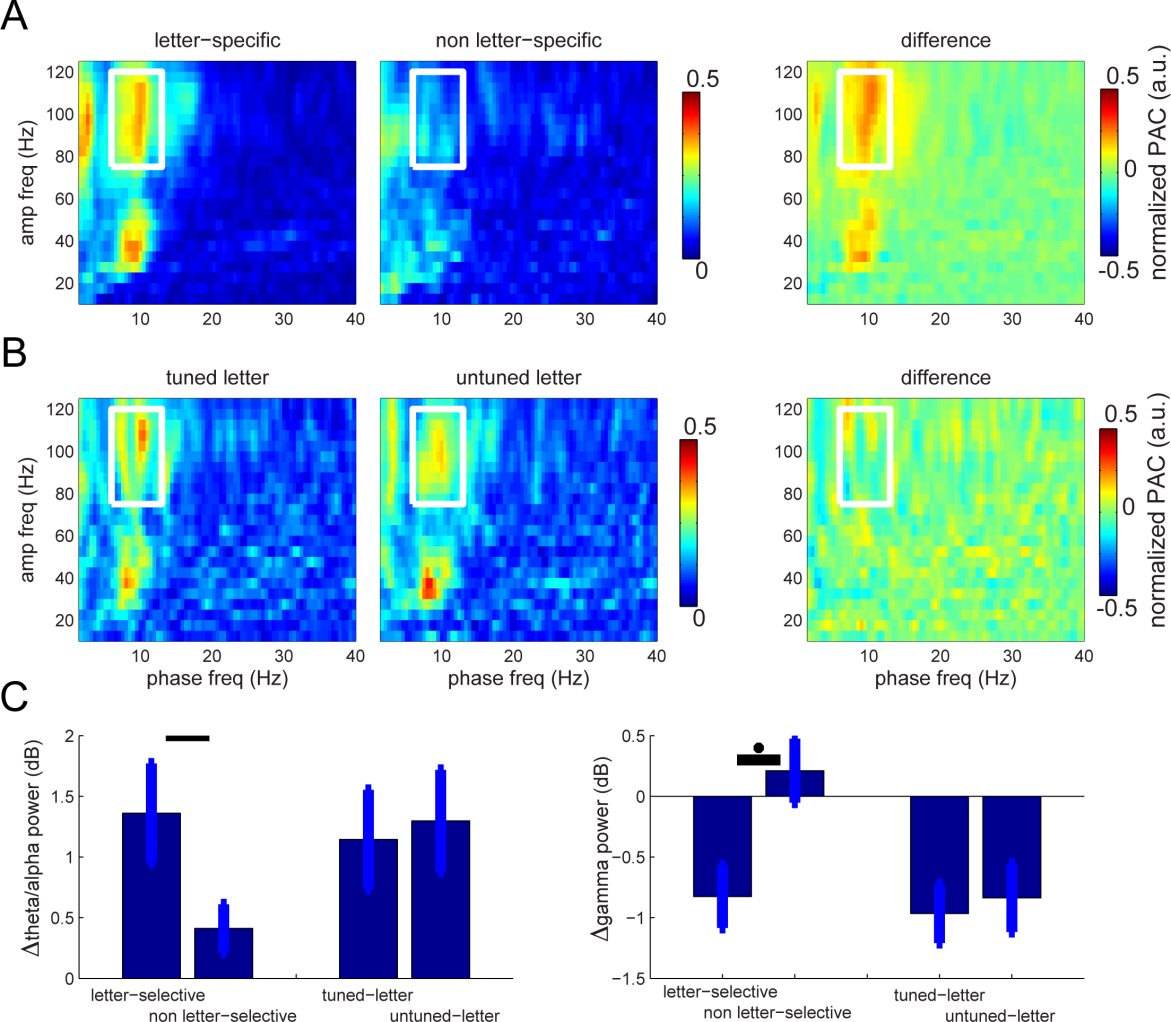
Properties of PAC during maintenance. (A) Average (14 sites) PAC is greater at letter-selective than at non-letter-selective sites. In right panel, differences were calculated for each subject; the average difference is large, showing that PAC is much larger at letter-selective sites (*p* < 0.012). (B) Average (14 sites) PAC is somewhat larger for tuned compared to untuned letters. In panel at right, difference for each subject was calculated before averaging. This difference was not statistically significant. (C) There is a significant difference (two-tailed *t* test; *p* = 0.012) of gamma power between letter-selective sites and non-letter-selective sites during the maintenance period. There is no significant difference between theta and gamma power over letter-selective sites when comparing the tuned letter and untuned letter during the maintenance period. Underlying data available at http://orion.bme.columbia.edu/jacobs/data/. a.u., arbitrary unit; PAC, phase–amplitude coupling

As shown in Figs 2A and 4C, this was not the case at letter-selective sites. Whereas theta/alpha power was greatly lowered during encoding, it increased significantly during maintenance compared to the baseline (*N* = 14; two-tailed *t* test *p* = 0.042). Gamma band power was low during maintenance. We note that similar effects to these were seen previously with electrocorticography (ECoG) [28], electroencephalography (EEG), and magnetoencephalography (MEG) recordings [29].

Previous work on human cortical oscillations has shown that theta–gamma coupling, a form of CFC, often occurs during cognitive tasks [21–23]. Because theta–gamma CFC is a central assumption of the LIJ model, we tested for this phenomenon, which occurred during the maintenance phase of the task. We did not calculate PAC during encoding because we were concerned about contamination from evoked activity. To quantify CFC, we used a measure of phase–amplitude coupling (PAC) that relates gamma band power to the phase of low-frequency theta oscillations [27,30] (see Methods). As mentioned before, Fig 3 shows the average normalized PAC over all selective and nonselective sites during maintenance, which we used to preselect the frequency band of interest. This plot revealed a peak in CFC between oscillatory phase in the theta/alpha band (7–13 Hz) and power in the high- (75–120 Hz) and low-gamma bands. We focused on the high-gamma band for our subsequent analyses rather than slow gamma (see Discussion) because we had already identified letter-specific activity in this range (see above), consistent with earlier work [14].

CFC during maintenance was greater at letter-selective sites compared to non-letter-selective sites (Effect size = 0.9, sample size *N* = 14; two-tailed *t* test *p* = 0.006; Fig 4A). Gamma power did not increase during maintenance but was coupled to the phase of theta/alpha oscillations. Notably, the fast gamma band that showed CFC was the same frequency range in which we observed letter-selective gamma elevation during encoding (Fig 2A; see also [21]). We next compared the magnitude of CFC between the maintenance of tuned versus untuned letters. While there was a small difference in CFC here for the theta/alpha by gamma frequency range, it was not significant (Fig 4B; see Methods). The mean levels of theta/alpha and gamma band power (Fig 4C right) were similar irrespective of whether a tuned letter was just viewed (*p* > 0.25). There was, however, a significant difference in theta/alpha and gamma power between letter-selective and non-letter-selective sites.

Recent work showed that nonsinusoidal waveforms can produce spurious CFC due to phase-specific high-frequency power increases from oscillatory harmonics (e.g., [31–33]. We examined this issue in our data by performing a control analysis in which we calculated the average traces locked to the theta/alpha peaks (S2 Fig). The resultant traces were largely sinusoidal, without sharp edges or triangular shapes. We therefore argue that the gamma activity observed in the PAC is not explained by asymmetry in the theta/alpha waveform. In summary, during WM maintenance, we found stronger PAC between the phase of alpha/theta oscillations and the power of the gamma band for letter-selective compared to non-letter-selective sites.

**Fig 5:**
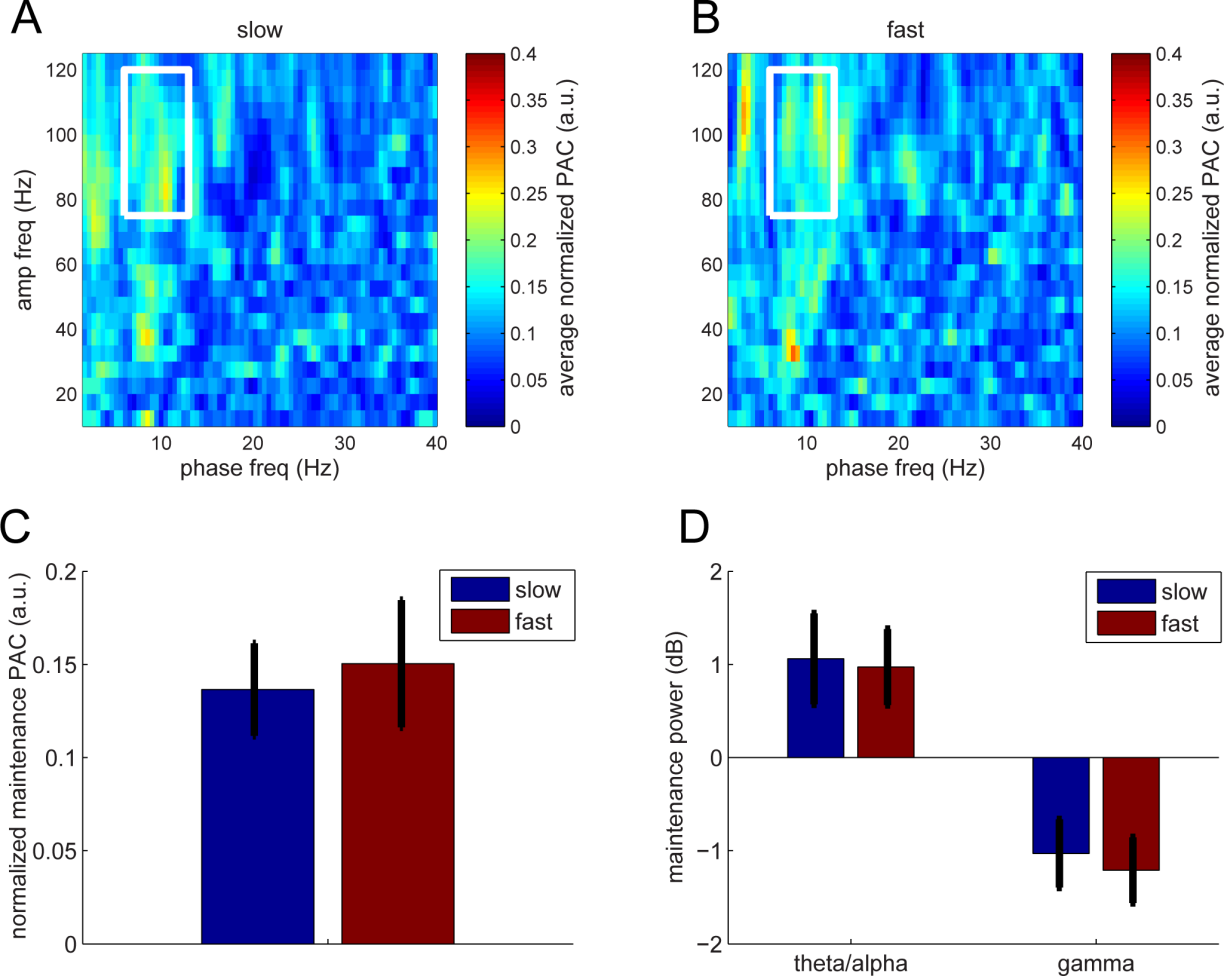
CFC during maintenance. (A,B) CFC in trials with slow or fast RT. (C) Average CFC (PAC) over the white box of A and B. There was no difference with respect to slow and fast RTs. (D) Theta/alpha and gamma power during maintenance. There was no difference with respect to fast and slow RTs. Error bars represent SEM. Underlying data available at http://orion.bme.columbia.edu/jacobs/data/. a.u., arbitrary unit; CFC, cross-frequency coupling; PAC, phase–amplitude coupling; RT, response time

Finally, we performed additional analyses to confirm that these effects were not driven by performance differences across subjects. We used reaction time to assess performance at the subject and trial level, using a median split to distinguish fast versus slow responses. As the hit rates were close to ceiling (94%), we did not perform a similar analysis on performance. There was no significant relationship between the magnitude of PAC during maintenance RTs over subjects (*p* > 0.3; *N* = 15; Fig 5) or between fast and slow trials from individual subjects. It should be noted that the LIJ model did not predict such a relationship either.

### Analysis of item maintenance according to theta/alpha phase

A key prediction of the LIJ model is that different memory items are represented by neuronal activity at distinct theta/alpha phases (Fig 1B). The broadband gamma signal that we measured is likely to reflect the spiking activity of individual neurons [34]. Thus, we predicted broadband gamma power at a letter-selective site would be elevated at the moment when a tuned letter is represented and, moreover, that the theta/alpha phase of maximum power would vary with the list position of a tuned letter (Fig 1B). We tested this prediction by measuring fast gamma band power (75–120 Hz) for tuned letters presented at the 3 list positions (P1, P2, and P3). For each site, we measured the position-specific deviation of fast broadband gamma band power from the overall average as a function of the local theta/alpha phase.

**Fig 6:**
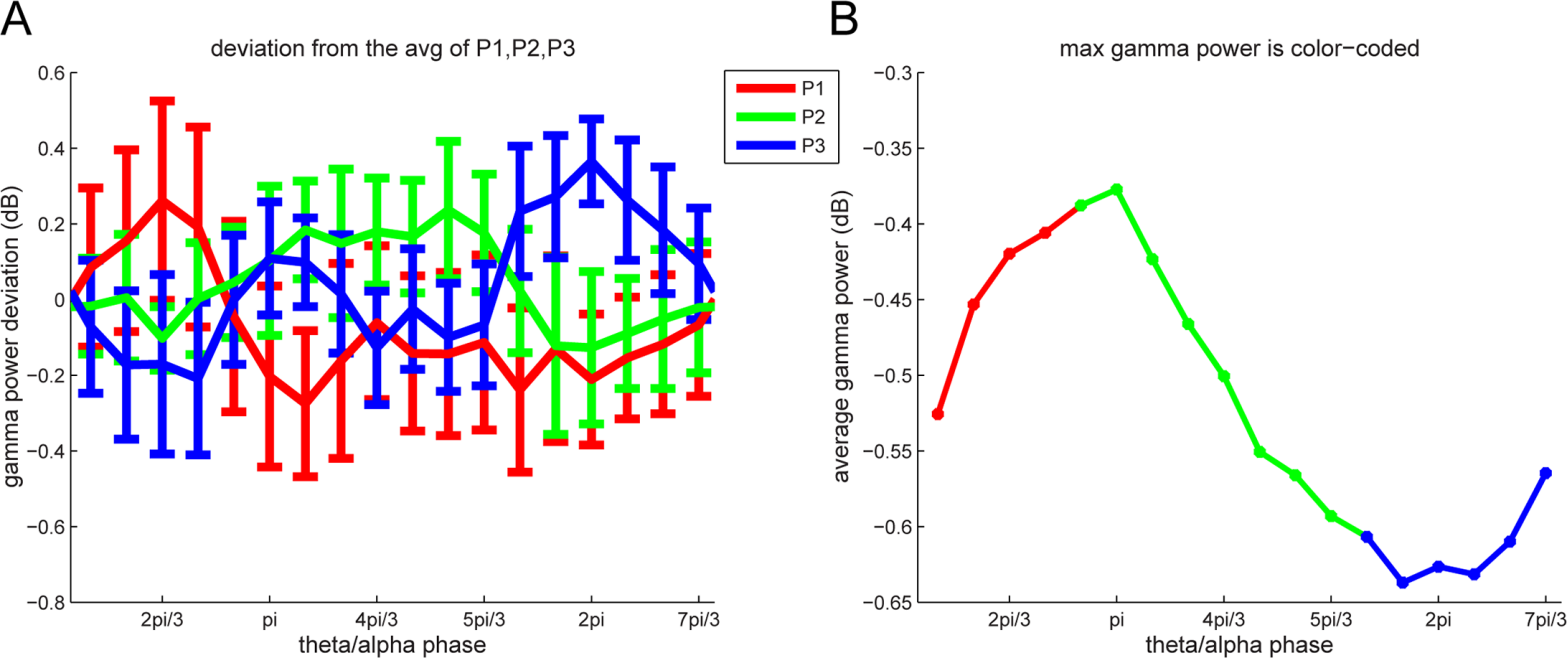
List position affects the theta/alpha phase that has maximal fast gamma power during maintenance. (A) The deviation of fast gamma band power for each position from the average measured as a function of theta/alpha phase (Hilbert phase 7–13 Hz). Error bars represent SEM. (B) Average fast gamma power as a function of theta/alpha phase; the color indicates the item position (P1-P2-P3) yielding the maximum power at that phase bin. We consider this sequence statistically significant in terms of ordering (*p* = 0.0062; see Methods). Underlying data available at http://orion.bme.columbia.edu/jacobs/data/.

As shown in Fig 6A, the preferred phase of the activation for tuned letters depended on the stimulus’s serial position in the list. Broadband gamma band power was higher at an earlier theta/alpha phase for items that were presented in P1 than for P2 and at an earlier phase for P2 than for P3 (see Fig 6A). A second way of analyzing these data, which confirmed this conclusion, is shown in Fig 6B. This figure shows the average fast gamma power as a function of theta/alpha phase over sites, with color indicating the item position that had the maximum power. Notably, here the color changes systematically with phase. Using a permutation test, we found that this pattern, consisting of 3 discrete clusters, was unlikely to have occurred by chance (*p* = 0.0062; see Methods). A similar phase dependence was not observed for slow gamma (*p* > 0.2; permutation test). Thus, item activation during memory maintenance, as measured by fast gamma power, has a theta/alpha phase that depends on the position in each list when the item was presented.

## Discussion

Historically, understanding human cortical WM processes has been complicated by the diversity of response patterns that appear across the cortex—we confirm that diversity here. To overcome this issue, a key step in our effort here to understand the neural basis of WM was our decision to focus on the cortical sites with demonstrable mnemonic content, as indicated by letter-selective gamma elevation [14]. By focusing our analyses on such sites, our results show that the amplitudes of oscillations are strongly affected by the task. Letter-selective sites showed specific oscillatory patterns during the encoding and maintenance phases of the task—most notably, increased high-frequency activity in the fast gamma band during encoding and, inversely, a decrease during maintenance. The opposite pattern was present for theta/alpha band activity. These gamma and theta/alpha patterns also interacted, which we observed as CFC between these signals during the maintenance phase of the task. The PAC during maintenance and the existence of letter-selective gamma activity allowed us to test the LIJ theory that the representation of items held in WM was supported by nested oscillations. Our analysis suggests that WM items are sequentially activated, as reflected by the fast gamma band activity within a theta/alpha cycle. The original model by Lisman and Idiart [12] suggested that theta oscillations are responsible for WM maintenance. We identified the effect to the 7–13 Hz range, which overlaps with both the theta and alpha band range. As a consequence, we refer to the theta/alpha band. It remains for future work to test if the theta and alpha band oscillations that fall at different ends of this range have different or similar functional roles in the human brain [35,36].

These results provide the strongest support to date for the serial organization of WM, as first proposed by Sternberg [9], and for the proposal (the LIJ model; [11,12]) that this organization depends on a gamma activity coupled to theta/alpha oscillations (Fig 1B).

### CFC during WM maintenance

CFC between gamma and theta oscillations is visible in the local field potential of the cortex and hippocampus in rodents and humans [28,37,38]. According to the LIJ model, these dual oscillations form a general coding scheme (the theta–gamma code; [11,16]), in which each item is represented by the spatial pattern of network activity that occurs during a gamma period within of a theta cycle (Fig 1B). While gamma power is relatively weak during the maintenance period; it reveals itself when related to theta/alpha phase. Given the complexity of cortical layers, the changes in total gamma amplitude could arise from many different mechanisms originating in different cell types with different memory functions during encoding and maintenance. The slow gamma power that we found during maintenance must be viewed in this light.

Evidence for temporal coding organized by oscillations came from the discovery of theta phase precession in hippocampal place cells [20] and the evidence that gamma oscillations subdivide the theta cycle into discrete phases [16,19,39]. Other work has shown that disruption or enhancement of theta–gamma oscillations can affect memory, which emphasizes the potential functional importance of this type of phase coding [40]. There is also evidence in humans supporting the importance of phase-based representations. Intracranial recordings in humans demonstrated that the hippocampus exhibits CFC during WM [37], and later work showed that during the encoding of item sequences, items are differentially modulated by theta phase in the human hippocampus according to their list position [17,41,42]. Together, these studies support the hypothesis that the hippocampus supports a theta–gamma code for episodic memory.

### A code for cortical WM maintenance organized by coupled oscillations

There has been slower progress in understanding the organization of a neuronal code by theta/alpha oscillations in neocortex [6,43]. It was shown that the locking of single neuronal firing to the theta rhythm predicted memory encoding in humans [44]. In neocortex, theta/alpha oscillations are generated by input from the thalamus and basal forebrain [45], slow gamma activity is locally generated by interaction of pyramidal cells and interneurons [46], and fast gamma activity is largely generated by spiking activity [47] (see also below). The existence of letter-selective sites in cortex has now made it possible to study temporal coding in more detail and to specifically test predictions of the LIJ model of WM (Fig 1B). The specific prediction of the LIJ model is that during maintenance, the fast-gamma power that reflects the neural representation of a stimulus should occur at a theta/alpha phase that depends on the item’s order in the list. The results shown in Fig 6 provide strong support for this prediction. This suggests that the brain’s mechanism to keeping multiple items active in WM is by a multiplexing mechanism in which different items activate serially at different phases of theta/alpha oscillations.

A second prediction of the LIJ model is that the serial organization of different memories is organized in time by ongoing gamma oscillations. This leads to the question of which type of gamma oscillation has this role. As shown in Fig 4A, left panel, CFC is present both in a broadband (fast gamma) range (70–120 Hz) and in a lower (slow gamma) range (30–50 Hz). These signals could, in fact, be related to each other: notably, broadband gamma correlates with neuronal spiking [10,34,48–53], spiking is phase locked to slow gamma [54,55], and fast gamma shows CFC with slow gamma [56,57]. Thus, the fact that fast gamma activity is item-specific (Fig 2A) and has a theta/alpha phase that depends on order (Fig 6) suggests that it reflects the spiking of cells that represent particular list items. It is notable that slow gamma power does not itself exhibit substantial letter selectivity (Fig 2A), because it suggests that slow gamma power does not carry information via amplitude but instead may temporally organize other neuronal signals, as hypothesized in Fig 1B.

### Relationship to behavioral results in the Sternberg task

The classic behavioral results on the Sternberg task were obtained in laboratory setting using well-trained subjects who were highly motivated to respond as quickly as possible. These results show that RT varies linearly with set size (S) and has an average slope of 38 ms for simple list items (letters or numbers) [9]. The exact properties of the RT distributions are known. The patients from whom we obtained brain recordings were neither trained nor highly motivated, and the experiments were done in a hospital setting. The RTs in these experiments thus were considerably slower than those obtained in the laboratory. However, it is nevertheless of interest to consider whether the underlying brain processes we have observed in patients might relate to the timing processes revealed in laboratory experiments.

The experiments in healthy subjects provided information not only on how the mean RT depends on S but also on how the standard deviation and skewness depends on S. These data could be accounted for by either of two computational models [58]⁠. In the “adapting theta model,” theta frequency was assumed to change with load, a change recently observed in the hippocampus [37]. The best fit of this model led to the prediction that the theta frequency for S = 3 is 8.3 Hz. This frequency is in good agreement with the approximately 9 Hz theta/alpha signal that we have observed experimentally. In the adapting theta model, the RT slope per item (38 msec) is 1.5–2 times the period of the underlying gamma oscillations. The model thus predicts a gamma frequency of 39–52 Hz, which is roughly consistent with the frequency range of slow gamma (30–50 Hz) that we observed with our CFC analysis (Fig 4). A second model, which assumes that stimuli reset the phase of theta [59,60], predicts similar frequencies of 7.2 Hz and 47 Hz for theta and gamma, respectively. Thus, these models, both of which are based solely on fits to RT distributions in the Sternberg task, make predictions about the frequencies that support WM that are concordant with our results from ECoG data. Following up our findings, it will be of interest to study how other manipulations, such as varying memory load, affect the properties of WM-related cortical oscillations to refine our understanding of how oscillations organize WM.

A successful theory of WM should also be able to explain the remarkable behavioral findings of Cavanagh [61]. He reported that the Sternberg slope increases with item complexity and, furthermore, that the number of items that could be remembered (span) decreases with item complexity. Remarkably, the product of span and slope remains constant. As argued in a recent review [62], the LIJ framework could explain this constancy if it assumed that span is determined by the number of gamma cycles in each theta cycle and that gamma period increases with item complexity. Recent physiological findings are relevant here. If complex items are represented by less sparse activity, as seems reasonable, then the known correlation between gamma period and mean neuronal activity [63] could explain why gamma period increases with complexity. Experiments could be conducted to test this relation, potentially providing a mechanistic basis for Cavanagh’s findings by directly demonstrating a link between WM span and gamma period. Although such efforts are in their early stage, there are notable successes [37,64–68].

In the future, it would of great interest to conduct a similar experiment to ours in which memory-load and memory content were each manipulated (e.g. [37]). This would allow for relating the individual theta/alpha and gamma frequencies to the “scan rate” of WM retrieval. Modulating the items’ complexity should be reflected in the gamma frequency. Finally, it should be mentioned that our results pertain to serial WM. It remains to be determined if similar oscillatory mechanisms are at play for multi-item WM when a serial order is not imposed by the stimulus presentation and if maintenance is still supported by a sequential coding scheme.

### Other models of WM

How does the evidence for a coupled oscillations model relate to other models that offer a different view of WM mechanisms? In the classic studies of single-unit activity during WM, firing persisted during the entire delay period [3]. However, recent findings indicate that activity can be discontinuous rather than continuous [8,69–72]. For example, sites in frontal cortex that have mnemonic activity show brief and sporadic bursts of spiking and gamma oscillations [73]. According to one theoretical framework [74], these bursts may depend on item-specific changes in synaptic weights that occur during encoding. If information was encoded in weights, it follows that persistent firing may not be necessary for the maintenance of stored information.

One possibility is that this evidence for discontinuous activity and the theta–gamma framework we proposed are in fact compatible and reflect the existence of dual memory mechanisms (probably in different regions), as suggested by psychological models that were based on behavioral data from normal and amnesic patients [75]. According to these models, items are first stored in a cortical limited-capacity buffer. Output from this buffer then drives synaptic modification in the high-capacity hippocampal network. Depending on conditions, recall of lists held in WM may depend on either the cortical buffer or the hippocampus. The network based on the LIJ model has the properties required to implement the buffer. It can rapidly store even novel information because information storage does not depend on synaptic modification, which can take seconds to develop [76]. Rather, in the LIJ model, memories are reactivated on every theta cycle by activity-dependent intrinsic conductances.

### Properties and function of low-frequency oscillations

Our results provide new information about the control of theta/alpha oscillations at content-specific cortical sites. As noted previously, various patterns of activity have been observed at sites not specifically linked to WM content (see also [23]). For instance, a previous study identified sites that undergo increases or decreases in theta power, changes that persist during both encoding and maintenance phases of the task [15]. Here, we show a quite different pattern at letter-selective sites—theta/alpha power goes down during encoding but up (even above baseline) during maintenance. The suppression may be related to the spatially nonuniform suppression of theta/alpha power that occurs in visual and sensorimotor cortices when a subregion is involved in a task [77,78].

We show here that during maintenance, many sites that show elevated theta/alpha power are nevertheless engaged in WM maintenance, as suggested by the fact that CFC is content-specific. Interestingly, the theta/alpha oscillations we observed largely resemble the conventional alpha oscillations observed in EEG and MEG recordings [29]: their amplitude is reduced during item presentation and increased during WM maintenance. In other ways, however, they seem to take on the role proposed for theta oscillations [11]. During maintenance, they seem to maintain a phase-organized code. We reconcile these findings by arguing that the reported theta/alpha oscillations gate external sensory information during item presentation while suppressing incoming input during maintenance. However, the same oscillations participate in an internal mechanism sustaining the WM representation during the maintenance interval. Consistently, we propose that theta/alpha generators in cortex and thalamus [79] inhibit the flow of incoming sensory information during maintenance but do not inhibit and indeed protect [80,81] the information that is already held in WM buffers. In future work, it would be interesting to directly manipulate the theta/alpha rhythm, for instance, with direct cortical stimulation to entrain or abolish the oscillations. This would serve to uncover the causal role of theta/alpha oscillations for WM maintenance, going beyond our current findings that did not yet demonstrate causality. It should be noted that our current analysis was constrained to the location of the intracranial electrodes. Studies based on EEG and MEG have identified cross-frequency interactions at related frequencies when quantifying neuronal dynamics at the large-scale network level [82]. In future work, it would be important to relate the phase-dependent effect we identified here to large-scale network dynamics.

### Conclusions

Although theta–gamma coupling appears in many brain regions [83], the generality of the theta–gamma code nonetheless remains unclear [11]. Early work established that this code organizes spatial information in the hippocampus [17,18,20,83], and recent work on the hippocampus has shown that it also organizes nonspatial information [17]. Here, by focusing on sites demonstrably engaged in WM, we showed that such coding occurs in cortex; specifically, the theta/alpha phase of item representation depends on item order during presentation. Furthermore, the estimates of theta/alpha and gamma frequencies that we obtained by measuring this phenomenon are in reasonable agreement with a model of RT distributions based on behavioral results. Thus, these results provide a coherent view of how the coupled alpha/theta and gamma oscillations could mediate WM. Taken together, these findings suggest that the theta/alpha phase code is a general neural coding scheme that helps represent ordered WM information in both neocortex and hippocampus.

## Methods

### Ethics statement

The research protocol was approved by Institutional Review Boards at the Hospital at the University of Pennsylvania (Philadelphia, PA, Protocol #802603) and the Thomas Jefferson University Hospital (Philadelphia, PA, Protocol #08F.464R) and adhered to the Declaration of Helsinki ethical guidelines. Informed written consent was obtained from each patient or their legal guardians.

### Recordings

Data of 15 epileptic patients (6 females, age: 36 ± 9) implanted with ECoG grids were included in this study. All subjects performed a Sternberg-type WM task [9] in which 3 letters were presented during encoding, and after 2 s of retention, a probe letter was presented either from the memory list or not. The details of the experiment and how data were collected have been previously reported in another paper [25], in which data from 15 patients were selected for inclusion based on sensor coverage and trial counts. The included data were selected at the beginning of this study prior to data analysis. Our reasons for excluding data from the earlier study [25] included the following: (1) Some data came from an unsuitable variant of the task with a short maintenance period. (2) The clinical recordings for some subjects contained transient interruptions due to file changes. (3) Some subjects had been recorded at a low sampling rate that precluded examining CFC. No data were collected during a seizure, and none of the analyzed sites with letter selectivity had been designated as sites where there had been seizure activity.

### Artifact rejection

We identified sites that were involved in seizure initiation, based on information provided by clinical teams. However, there were still remaining electrodes with occasional epileptiform discharges. To reduce their impact, we rejected epochs for which such activity was present. We used kurtosis to identify EEG segments with these epileptiform discharges [84–87], by computing the kurtosis for each individual EEG segment and labeling any segments with kurtosis above 5 as being potentially epileptogenic. Visual inspection confirmed that this approach worked correctly, because these periods, although rare, contained unusual EEG such as epileptic spikes or sharp waves. For more detail on this method, see [87]. On average, we used 78% ± 5% of all trials, which amounted to 67 ± 12 trials (the number of trials included/excluded for the 14 sites were 104/2, 42/10, 174/3, 47/11, 41/5, 49/6, 33/7, 49/14, 24/15, 47/8, 77/12, 73/9, 104/4, 107/1).

### Preprocessing

All recordings were resampled to 250 Hz. Furthermore, as commonly done [14,88,89], we re-referenced the data to the common average over all ECoG electrodes. We chose this method because it previously proved useful for simultaneously identifying both theta and gamma oscillations [14], rather than a different approach such as bipolar referencing, which might have changed the spatial characteristics of the signals. Alternatively, the analysis could have been done using the scalp electrodes as reference; however, such procedure could have made the results difficult to interpret because the scalp electrodes are sensitive to posterior alpha rhythm. Line noise was removed by using discrete Fourier transform with filtering centered at 60 and 120 Hz.

### Time-frequency analysis

Time-frequency analysis of phase and power was performed using a sliding window FFT approach. To estimate the power, we used sliding time windows (50 ms steps) that varied with frequency (dT = 3/f; i.e., 3 cycles long) for power estimates from 1 to 30 Hz in steps of 1 Hz. For power estimates from 30 to 125 Hz, we used a fixed-length time window (dT = 100 ms; in steps of 5 Hz). The analysis was done in 1–7 s intervals relative to the first letter onset. Prior to the FFT, the windows were multiplied with a Hanning window of equal length.

For each trial, the power in the baseline (−0.8 to −0.35 s before first letter presentation) was calculated as the average power. The encoding period was defined as 0.2–0.5 s relative to the onset of each letter. The maintenance period was defined as −1.85 to −0.3 s relative to the probe onset.

### Letter-selective sites

A primary goal of our statistical analyses was to identify electrodes in which the gamma power was systematically different for different letters [14]. The letter specificity of each individual electrode was identified from analyses of data from the encoding period. Subsequently, we used this information to inform phase and PAC analyses that examined data from the maintenance interval. We used MI to measure the dependence between gamma power and letter [90]. This procedure identified sites that could distinguish between two letters X and Y in terms of their resulting gamma power response. In the case of a subject who viewed 16 different letters across the task, there were 16 × 15/2 = 120 pairs to be tested for each electrode. High MI between gamma power and labels consistent over trials would suggest a high selectivity in terms of gamma power.

We computed this measure as MI([*γ*_X_, *γ*_Y_];[0_nx_,1_ny_]) = H([*γ*_X_, *γ*_Y_]) − H([*γ*_X_,*γ*_Y_]|[0_nx_,1_ny_]), where H is the entropy function, and nx and ny refer to the number of trials for X and Y, respectively. Specifically, entropy was calculated as p(x)log(p(x)). The probability p(*γ*) was calculated by dividing the gamma power into 256 equal bins and then estimating a distribution over the number of occurrences of each power bin. The vector [*γ*_X_,*γ*_Y_] is the baseline-corrected gamma power (70–100 Hz), and [0_nx_,1_ny_] is a binary vector where *0* represents for the first letter and *1* the second. For the statistical assessment, a null distribution was made by permuting the class labels (all possible combinations) and then recalculating the MI. The *p*-value corresponded to the fraction of MI values from the permutation distribution that were above the actual MI to be tested. We Bonferroni-corrected this *p*-value for the number of electrodes and 120 letter pairs. The procedure allowed us to identify sites with significant selectivity after correction with respect to *p* < 0.05 in 14 independent sites in 9 (of 15 subjects); 1 subject had 3 sites, and 3 subjects had 2 sites.

For each electrode, the pair of letters that resulted in the highest MI was selected, and the one with the higher gamma power was termed the tuned letter, and the other one was termed the untuned letter. Therefore, when a tuned letter was presented to the patient, the relevant letter-selective site exhibited a relative increase in gamma power during encoding. To examine letter tuning of content-specific sites during maintenance, for the tuned letters, we only considered trials in which at least one of the maintained items was the tuned letter and in which the untuned letter was not presented. Inversely, for examining the maintenance of untuned letters at these sites, we considered trials in which at least one of the presented items was the untuned letter and in which none of them was the tuned letter.

### CFC

PAC was calculated as the coherence between low-frequency oscillations and the envelope of high-frequency activity [27,91]. For each trial, the power envelope of the high-frequency activity was estimated from the FFT of a frequency-dependent sliding time window (6 cycles long; i.e., dT = 6/f) multiplied by Hanning taper. This was done from 10 to 125 Hz in steps of 5 Hz. The PAC was estimated as the coherence between this envelope and the raw signal. The 2 s of the retention interval was divided into 3 segments of 1 s (centered at 0.5 s, 1.0 s, and 1.5 s in the retention interval; i.e., 50% overlap). The coherence was first calculated in each of these segments individually and then averaged over segments and trials.

When comparing the PAC measures between conditions (letter-specific versus non-letter-specific sites; tuned versus untuned), the values were averaged in frequency-by-frequency regions of interest (7–13 Hz by 75–120 Hz; see Fig 3). The frequency ranges were obtained from the PAC combined over all electrodes and trials. These are consistent with the dominant frequency modulations in Fig 2. To compare PAC over sites, the average PAC values were compared using a paired *t* test for the frequencies of interest.

### CFC normalization for visualization

The PAC representations were normalized per electrode in the shown grand average (Fig 3). Here, we computed the complete PAC for each electrode, then normalized each individual PAC spectrum by dividing by the electrode’s maximum PAC value, and finally averaged the normalized PAC spectra over sites.

### Permutation test for serial ordering

We conducted a permutation procedure to assess the statistical reliability of the observation that the gamma band representations of individual letters systematically occurred at distinct, sequential theta-phase bins according to the position of the presented item in the list (Fig 6). This procedure used a template-matching approach, in which we computed a fit statistic that measured the similarity between the observed gamma power distribution and our hypothesized templates. We then computed this fit separately for the observed data and for 10,000 surrogate datasets, obtained by randomly shuffling the original data. This shuffling was performed by changing only the trial label of each event, thus, critically, allowing us to assess significance while preserving the temporal and spectral structure of the underlying neural data.

For the real and shuffled datasets, on each electrode, we computed instantaneous theta/alpha phase and gamma power during the maintenance period of each trial using the Hilbert transform, filtering at 7–13 Hz and 75–120 Hz, respectively. We smoothed the measure of gamma power using a 40 ms Hanning-tapered sliding window. Then, we divided the theta cycles into 18 equal-sized phase bins (spanning 0 to 2π radians) and computed the mean fast-gamma power for each bin. Then, to aggregate the results, we averaged the results of this calculation across trials when the tuned letter was presented in each of the first (P1), second (P2), and third (P3) positions and then averaged across electrodes. Finally, we converted this gamma power distribution into a set of 18 labels (one for each theta phase bin), each of which indicates the list position that was represented in a given bin (i.e., had the highest gamma power).

Our primary hypothesis was that the gamma representations of individual list positions occurred at distinct, monotonically increasing theta-phase bins. We measured the similarity of a dataset to this pattern by first creating a template in which the 18 phase bins were split equally to each represent the 3 list positions in 3 contiguous ordered groups. This template contained 6 ones, followed by 6 twos, and then 6 threes. To avoid assumptions about a specific absolute theta phase in which the representation of a list began, 17 additional templates were created that were circular rotations of the template. The fit score for each observed gamma power distribution was the count of the number of bins in which the list position label matched the template’s corresponding label. Each distribution’s score was taken as the maximum fit score across all 18 templates. This entire approach was repeated by computing the fit scores of surrogate gamma power distributions that had been computed by shuffling. Finally, we assessed statistical significance by measuring the rank (*p*-value) of the actual gamma power distribution relative to the distribution of surrogate template fits. The *p*-value from this procedure indicates the proportion of surrogate data that better or equally fit the template than the actual dataset. Thus, a small *p*-value indicates that we can reject the null hypothesis that shuffled data were likely to produce the monotonic sequential gamma item distributions we observed (Fig 6).

## Supporting information

S1 Table Location of letter-specific sites. (DOCX)

S1 Fig Demonstration that data from Fig 2A are reproducible over the 14 letter-selective sites of 9 subjects. Time-frequency representation of power of the most selective and the least selective sites (highest and lowest mutual information) with their corresponding letters over 9 subjects. Average baseline power is subtracted from all time points. Subject number and location of electrodes are shown above each plot. The segments between white lines should not be interpreted as letter order. During different segments, letters with different tuning are presented. Underlying data available at http://orion.bme.columbia.edu/jacobs/data/. E, encoding; M, maintenance; P, probe.
(TIF)

S2 Fig Epochs (approximately 300 ms) of the data were aligned according to the peak in the theta/alpha band and averaged (black lines). The peaks were identified from the data filtered at 7–13 Hz; however, lower traces (black lines) were the resulting peak-locked averaged from the unfiltered data. The time-frequency representations of power were calculated for the phase-aligned epochs and averaged as well. We observed a clear modulation in the gamma band with respect to the phase of the theta/alpha oscillations. Note that the averaged traces were largely sinusoidal in shape; i.e., the modulations in the gamma band are not likely to be explained by harmonic contributions from nonsinusoidal wave shapes. Furthermore, the high-frequency modulation is primarily constrained to the gamma band (nonsinusoidal effects will be visible in the full frequency range). Underlying data available at http://orion.bme.columbia.edu/jacobs/data/.
(TIF)

## Abbreviations

a.u.: arbitrary unit
CFC: cross-frequency coupling
ECoG: electrocorticography
EEG: electroencephalography
LIJ: Lisman/Idiart/Jensen
LTM: long-term memory
MEG: magnetoencephalography
MI: mutual information
PAC: phase–amplitude coupling
RT: response time
S: set size
TFR: time-frequency representation
WM: working memory
