# Supplementary material for: Serial representation of items during working memory maintenance at letter-selective cortical sites
Source: PLoS Biol. 2018 Aug 15;16(8):e2003805. doi: 10.1371/journal.pbio.2003805 (PMC6093599; doi:10.1371/journal.pbio.2003805)
Supplement: S1 Table — (DOCX) [file pbio.2003805.s001.docx]

***S1 Table:*** *Location of letter specific sites.*

| **subject** | **Location** |
| --- | --- |
| 1 | 2 x middle temporal gyrus, superior parietal lobule |
| 2 | Fusiform gyrus |
| 3 | Fusiform gyrus, middle temporal gyrus |
| 4 | Inferior occipital gyrus, Middle occipital gyrus |
| 5 | Inferior temporal gyrus |
| 6 | 2 x Fusiform gyrus |
| 7 | Fusiform gyrus |
| 8 | Fusiform gyrus |
| 9 | Superior temporal gyrus |
