# Supplementary figures and images for: Serial representation of items during working memory maintenance at letter-selective cortical sites
Source: PLoS Biol. 2018 Aug 15;16(8):e2003805. doi: 10.1371/journal.pbio.2003805 (PMC6093599; doi:10.1371/journal.pbio.2003805)

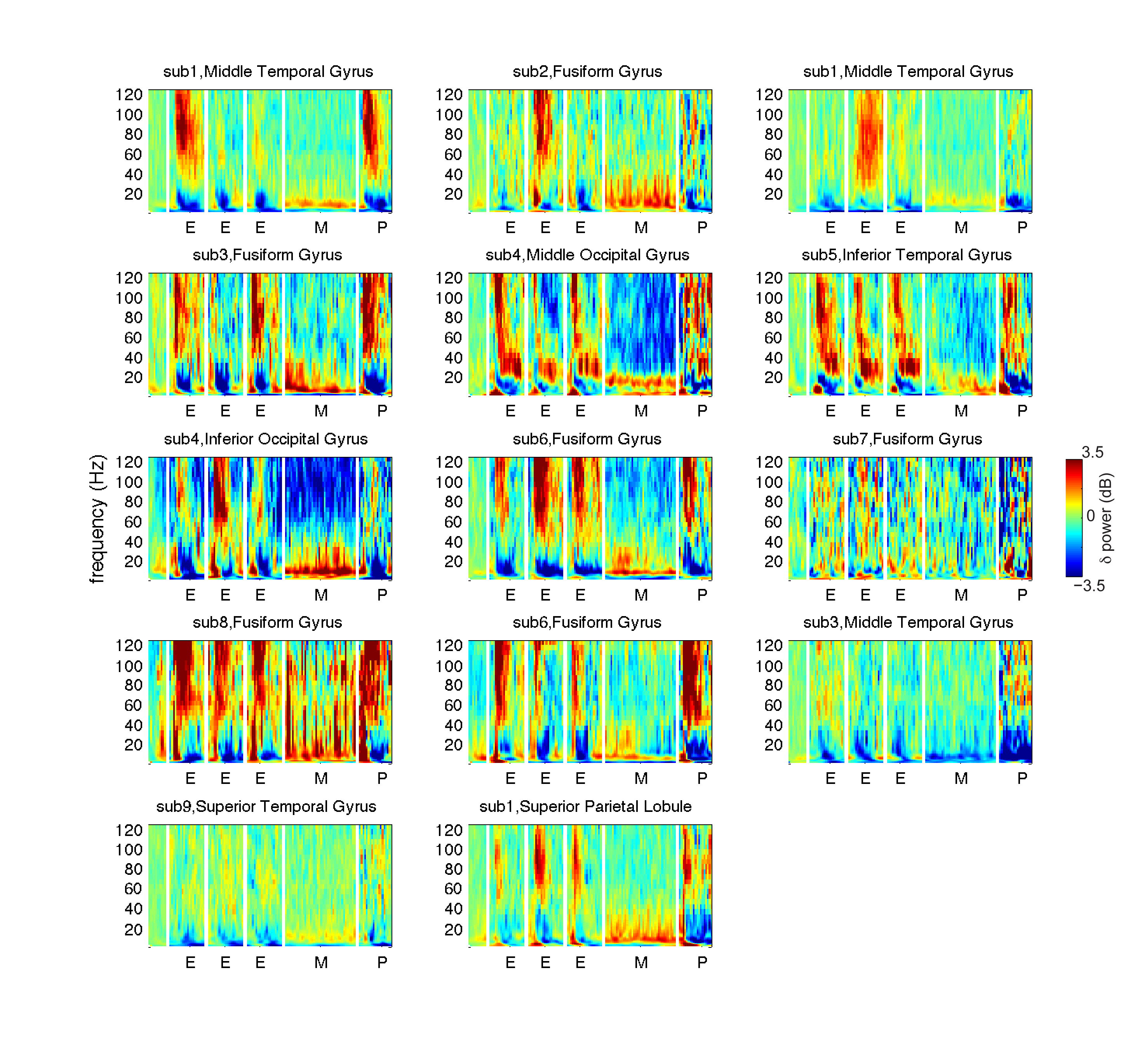

Supplement: S1 Fig — Time-frequency representation of power of the most selective and the least selective sites (highest and lowest mutual information) with their corresponding letters over 9 subjects. Average baseline power is subtracted from all time points. Subject number and location of electrodes are shown above each plot. The segments between white lines should not be interpreted as letter order. During different segments, letters with different tuning are presented. Underlying data available at http://orion.bme.columbia.edu/jacobs/data/. E, encoding; M, maintenance; P, probe. (TIF) [file pbio.2003805.s002.tif]

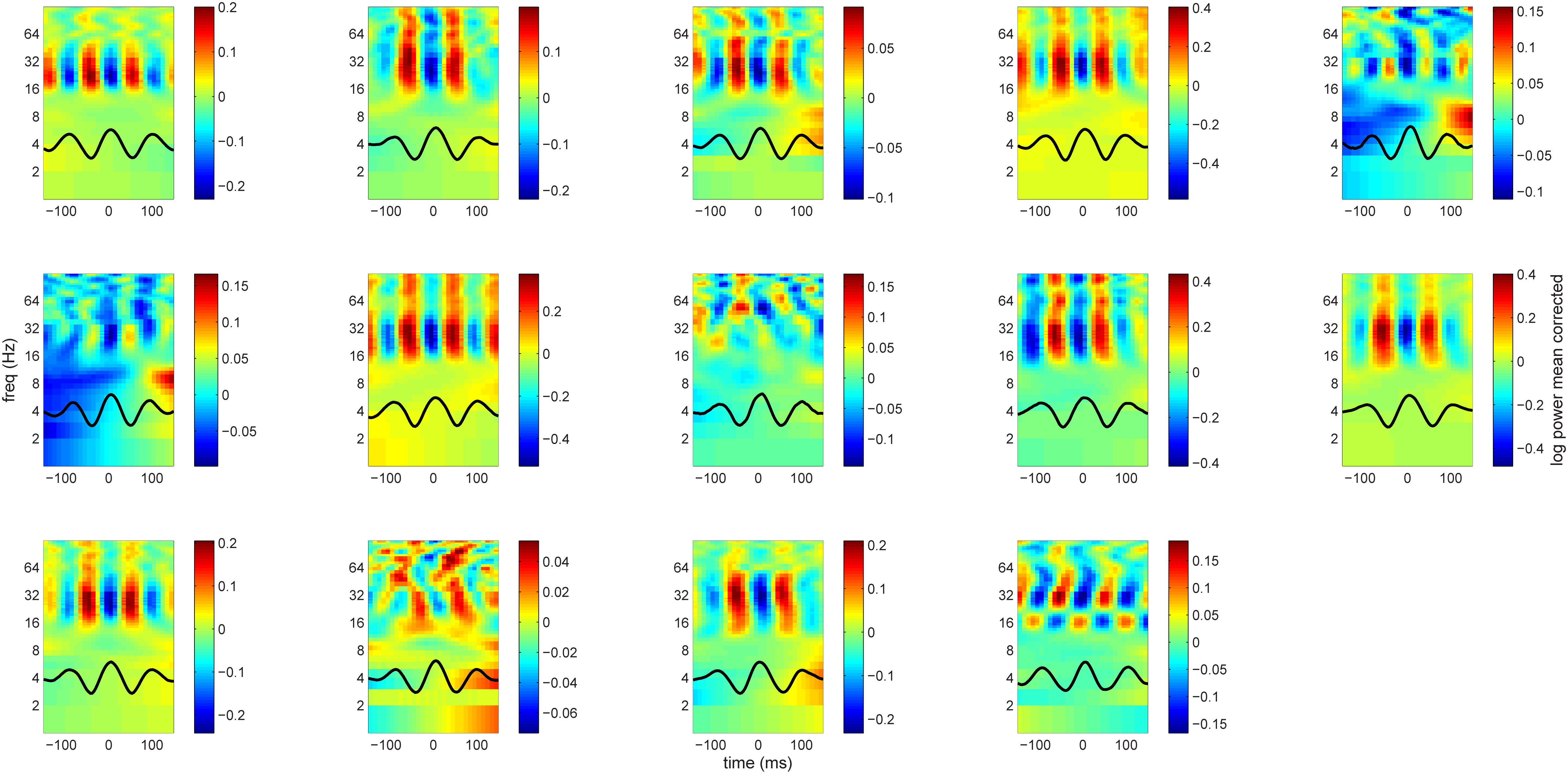

Supplement: S2 Fig — The peaks were identified from the data filtered at 7–13 Hz; however, lower traces (black lines) were the resulting peak-locked averaged from the unfiltered data. The time-frequency representations of power were calculated for the phase-aligned epochs and averaged as well. We observed a clear modulation in the gamma band with respect to the phase of the theta/alpha oscillations. Note that the averaged traces were largely sinusoidal in shape; i.e., the modulations in the gamma band are not likely to be explained by harmonic contributions from nonsinusoidal wave shapes. Furthermore, the high-frequency modulation is primarily constrained to the gamma band (nonsinusoidal effects will be visible in the full frequency range). Underlying data available at http://orion.bme.columbia.edu/jacobs/data/. (TIF) [file pbio.2003805.s003.tif]
